# Preparation of N-Halamine Gelatin Sponge and Its Application in the Treatment of Skin Infection

**DOI:** 10.3390/polym16182579

**Published:** 2024-09-12

**Authors:** Jiahao Zhu, Jiageng Xue, Huaiying Qin, Yiqing Wang, Yefan Wang, Yidan Cheng, Yingxia Ma, Xiaoyun Zhang, Chenliang Gong, Guanghui Zhao

**Affiliations:** 1 State Key Laboratory of Applied Organic Chemistry, Institute of Biochemical Engineering & Environmental Technology, College of Chemistry and Chemical Engineering, Lanzhou University, Lanzhou 730000, China; 2 School of Materials Science and Engineering, Lanzhou University of Technology, Lanzhou 730030, China; 3 School of Pharmacy, Lanzhou University, Lanzhou 730000, China

**Keywords:** N-halamines, natural macromolecule, antibacterial, biomedical

## Abstract

Nowadays, there has been an increasing research interest into N-halamine compounds due to their wide antimicrobial properties and no drug resistance. Most of the research mainly focuses on small molecular N-halamines, while few studies are on macromolecule N-halamines. In this work, antibacterial N-halamine polymer materials based on proteins (GS-Cl) were synthesized with an antibacterial component of oxidative chlorine, a support component of a gelatin sponge. After carrying out systematic characterization, the GS-Cls exhibited well-defined porous morphology and had a high efficiency in the killing of Gram-positive bacteria (*E. coli*) and Gram-negative bacteria (*S. aureus*). The loading of oxidative chlorine (Cl^+^%) could be controlled by changing the NaClO concentrations and chlorination times. The biocompatibility was confirmed as well. In vivo experiments suggested that the GS-Cl sample could effectively promote the healing of skin wounds in mice *E. coli* and *S. aureus* infection models. These studies show that proteins can be chlorinated and endowed with antimicrobial properties, which has great application potential in the treatment of bacteria-infected wounds.

## 1. Introduction

The skin, as an important defense organ, plays a fundamental role in defending both external physical and internal chemical damage [1,2]. However, the skin is extremely vulnerable in life, and skin damage caused by surgery is inevitable [3,4]. Once skin tissue damage occurs, the skin will lose the microbial infection of the wound site, the most basic protective effect, causing severe inflammation of the wound, inhibiting wound healing, and even leading to infection-related syndrome [5], which eventually becomes one of the important factors threatening human health and people’s lives [6].

Although antibiotics have been widely used to treat bacteria-infected wounds, chronic overuse and misuse of antibiotics have promoted the development of antibiotic-resistant bacteria, gradually increasing the mortality caused by multidrug-resistant bacterial infections [7], which has become a major threat to human and public health [8,9]. Nano-silver (SNP) [10], biguanides [10,11], chitosan [12], N-halogenamine [13], and other antibacterial agents that have been developed have certain antibacterial effects on bacterial infection wounds. However, researchers have found that applying SNP to wounds can cause an accumulation of silver and toxicity to a variety of cell lines, including to lung, kidney, brain, and endothelial cells [14]. Several other studies reported the cytotoxic effects of poly hexamethylene biguanide (PHMB) on chondrocytes, endothelial cells, and osteoblasts [15,16]. One study reported that there were few antibacterial properties of chitosan under neutral pH limits application [17]. Recently, there has been increasing research interest into N-halamine compounds due to their wide antimicrobial properties and no drug resistance [18].

N-halamines are compounds containing N-Cl, N-Br, or N-I covalent bond(s) that are obtained by the halogenation of N-H groups [19]. Its antibacterial activity is due to the positive charge of halogen atoms in the N-Cl, N-Br, or N-I bond, which is oxidizing. N-halamines could release strong oxidizing halogen cations in water and straightly react with the bacteria, destroy cell membranes, enter the microbial body, and interfere with cellular enzyme activity and metabolic processes, resulting in bacterial death [20]. According to the different halogenation groups, N-halamines could be divided into three types, namely amine, amide, and imide N-halamines [21]. In aqueous solutions, the structural stabilities of these three categories are found that in the order of amine, amide, and imide N-halamines due to increasing dissociation constant [22]. On the contrary, their bactericidal activities follow a totally opposite tendency. During the overall consideration, it indicates that amine and amide N-halamines could be used as an N-halamine precursor. At present, domestic and foreign scholars have studied various applications of N-halamines, including water disinfection [23], air purification [24], and textile products [25]. And the research in biomedical applications, particularly in wound therapy, is insufficient until now. Some researchers prepared N-halamine materials by physical modification. As such, Zheng et al. [26] prepared a novel chitosan (CS) sponge with cross-linker 1,2-epoxy-4-vinyl cyclohexane (EVC). An antibacterial CS/EVC/MC sponge was prepared by immersing it in a 1-chloro-2,2,5,5-tetramethyl-4-imidazolinone (MC) solution. The results of the antibacterial experiment revealed that the CS/EVC/MC sponge could kill approximately six logs of *S. aureus* and *E. coli* within 10 and 30 min, respectively. Furthermore, the cytotoxicity test indicated that the CS/EVC/MC had biocompatibility, which suggests that the sponge has biosafety in wound treatment. But, the method still has problems, such as that antibacterial activities would decline, induced by the leach of low-molecular N-halamines. Several studies suggested that chemical modification with covalent linkage can effectively incorporate the N-halamine moieties into materials. For instance, Wu et al. [27] designed and constructed a porous N-halamine polymeric coating on the titanium surface. The antibacterial experiments showed that Ti-PAA-NCL had strong antibacterial activity against *S. aureus* and *P. gingivalis*, with antibacterial rates of 96% and 91%. While this approach does improve durability, the process may be complicated. Protein is one of the most important natural macromolecules and is an essential component of human cells and tissues [28,29]. Due to its characteristics of biocompatibility, biodegradability, and low cytotoxicity, there is a great application prospect in the nanomedicine field, including wound healing [30,31]. Proteins have multifunctional groups such as amides and amines, so they have the potential to be chlorinated and have antibacterial properties. Given the structure and properties of proteins and the advantages of N-chlorinated amines, it is often a simpler approach to directly utilize proteins containing N-H bonds.

**Scheme 1:**
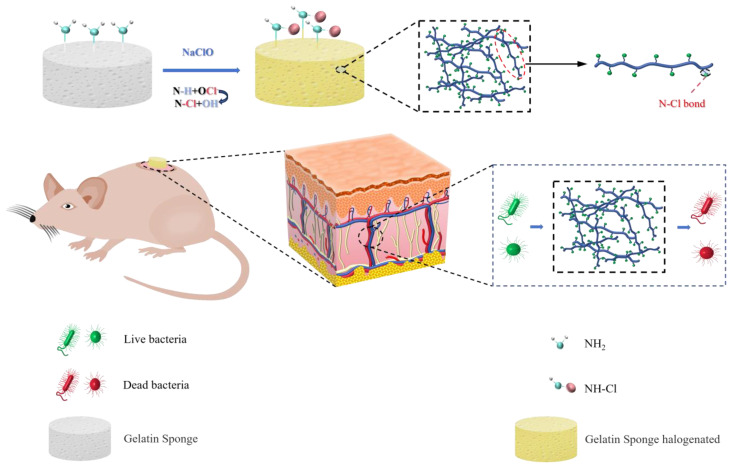
Synthesis and antibacterial mechanism of chlorinated gelatin sponge.

In this study, we prepare N-halamines of proteins for antibacterial applications by a super-convenient strategy, using a gelatin sponge as an N-halamine model with N-Cl bond. As shown in Scheme 1, a chlorinated gelatin sponge (GS-Cl) was prepared by a one-step N-chlorination reaction. Up to now, proteins possess abundant amide and amine groups and have been used as an N-halamine precursor, rendering great prospects in N-halamine wound therapy. After a series of adjustments under synthetic conditions, gelatine sponges with different active chlorine contents were obtained. In in vitro tests, the GS-Cl can effectively kill both *S. aureus* and *E. coli* based on the antibacterial property of the N-Cl bond. The wound-healing property of GS-Cl was evaluated by an in vivo test of *a* skin wound infection model of mice. These studies demonstrated that proteins can be chlorinated and endowed with antibacterial properties, which has great application potential in bacterial infection wound treatment.

## 2. Experimental Section

### 2.1. Materials

Sodium hypochlorite, sodium thiosulfate, and potassium iodide were obtained from Tianjin Damao Chemical Reagent Factory. Starch was obtained from the Tianjin Guangfu Fifine Chemical Research Institute. The medical gelatin sponge was provided by Xiang’en Medical Technology Development Co., Ltd. (Nanchang China). Deionized water, from a water purification system (Millipore system) was used throughout the experimental work.

### 2.2. Measurements

The Fourier-transform infrared spectrometer (FTIR) recorded on an Affinity. The X-ray photoelectron spectroscopy (XPS) was recorded on a Kratos AXIS Ultra DLD (Beijing China). Scanning electron microscopy (SEM) was measured on a Hitachi S-4800 (Qingdao China). The tensile tests were measured on a tensile machine (AGX-V 500N, Shanghai, China)

All animal experiments were conducted in compliance with the Animal Ethical Procedures and Guidelines of the People’s Republic of China. All experiments were conducted in compliance with the Animal Ethical Procedures and Guidelines of the People’s Republic of China and were approved by the Animal Ethics Committee of Lanzhou University (China).

### 2.3. Synthesis of Protein N-Halamines

Protein N-halamines for wound therapy applications were prepared using a super-convenient strategy with a gelatin sponge as a typical example. In order to prepare the gelatin sponge wound dressing (GS-Cl) with different active chlorine content, an N-chlorinating agent (sodium hypochlorite) was diluted into 50 mL aqueous solutions with different active chlorine contents (0.03%, 0.05%, 0.1%, and 0.3%). Then, the solution was adjusted by 1 N H_2_SO_4_ to pH = 7, and GS was added into the solution, stirred for 2 h at room temperature, and then repeatedly rinsed with water to remove the remaining NaClO. The sponge was frozen in a freeze-drying device for 48 h to obtain the final GS-Cl sample. The freeze-dying device had a vacuum below 40 Pa and a temperature below −45 °C. And the sample was stored in a desiccator at 25 °C before further tests.

### 2.4. Mechanical Properties Test

The mechanical properties of the GS and GS-Cl samples were evaluated with 100 mm min^−1^ tensile velocity, and the samples were cut into the same long strip shape. The Young’s modulus was calculated by the slope (4–10% strain), and the toughness was calculated by the integral area of the stress–strain curve.

### 2.5. Characterization

Qualitative analysis of the GS and GS-Cl were given by X-ray photoelectron spectroscopy (XPS) PHI5702 (Beijing China), Al Kα (1486.7 eV) inc with monochromatic. Infrared spectrum data was recorded by a Fourier-transform infrared spectrometer (FTIR, nexus 670, Madison, WI, USA). The morphologies were characterized by SEM at 5.0 KV accelerating voltage.

### 2.6. Determination of GS-Cl Samples’ Chlorine Content

The iodine/thiosulfate titration method was used to determine the loading of active chlorine of the GS-Cl samples. To be brief, 0.1 g GS-Cl samples were immersed in 10 mL of water with 0.2 g KI in a beaker. Then, 5 mL of 2 mol L^−1^H_2_SO_4_ were added. After stirring, the color became blue when adding a drop of 10% *w*/*v* starch solution. Then, sodium thiosulfate (Na_2_S_2_O_3_) was used to titrate the solution, and the end point was determined by the color change. The *Cl^+^* (%) in the GS-Cl samples was calculated by Equation (1) below:

(1) $${Cl}^{+}\left(\%\right)=\frac{C\times V\times M}{2m}\times 100\%$$

where *C* represents the molar concentration (mol L^−1^) of the Na_2_S_2_O_3_, *V* represents the volume (L) of that consumed, *M* is the relative molecular mass (g mol^−1^) of *Cl*, and *m* is the weight (g) of the GS-Cl samples. 

The rinsed water was also determined by the iodine/thiosulfate titration method until there was no Cl^+^ left to make sure the remaining NaClO was completely removed.

### 2.7. In Vitro Antibacterial Test

The plate-counting method and agar diffusion test were carried out in vitro antibacterial tests. *E. coli* and *S. aureus* grew in liquid LB broth for 12 h at 37 °C with shaking. Then, bacteria were diluted to suspension (2 × 10^8^ CFU mL^−1^) with nutrient broth. In the plate-counting method, samples were placed in a 48-well plate and immersed in 300 μL of *S. aureus* or *E. coli* suspension (2 × 10^8^ CFU mL^−1^). Then, they were incubated for 12 h at 37 °C. The above bacterial solution was diluted with the nutrient medium 10,000 times. Then 100 μL diluted bacterial solution were spread on agar plates. Then, they were incubated for 12 h, and the colonies were observed. Antibacterial rates were calculated by Equation (2) below:

(2) $$Antibacterial\;rate=\left(1-\frac{B}{A}\right)\times 100\%$$

where the bacterial solution and the nutrient medium were selected as positive and negative contrasts, respectively, A is the number of bacteria in the control group, and B is the number of bacteria in GS-Cl. In the inhibition zone study, 100 μL bacteria suspensions (2 × 10^8^ CFU mL^−1^) were dispersed onto solid LB agar plates. Then, pour 50 μL of PBS buffer solution into gelatin sponges. The different gelatin sponges, with a diameter of 10 mm, were put on the plates. Then, after the plates were incubated for 12 h at 37 °C, the width of the inhibition zones was calculated by Equation (3) below:

(3) $$Width=\frac{D-d}{2}$$

Before the SEM test, the bacteria were fixed with a 2.5% glutaraldehyde solution for 12 h and then gradually dehydrated by a series of ethanol solutions (20%, 40%, 60%, 80%, and 100%).

A live/dead bacterial test was performed to examine the viability of the bacteria. Samples were placed in a 48-well plate, immersed in 300 μL of *S. aureus* or *E. coli* suspension (2 × 10^8^ CFU mL^−1^), and incubated at 37 °C for 12 h. Then, the bacteria were centrifuged at 5000 rpm for 5 min and stained for 30 min with a SYTO 9/propidium iodide (PI) reagent kit in the dark at 37 °C. Then, the bacteria were washed with PBS (pH = 7.4) and were dispersed in 1 mL of PBS (pH = 7.4). Then, they were imaged by inverted fluorescence microscopy.

### 2.8. Biocompatibility Assay

Biocompatibility of the GS-Cl sample was evaluated by using human keratinocytes (HaCaT) with the CCK-8 assay in this study. First, HaCat cells were placed with a density of 6 × 10^4^ cells/well in a 96-well plate. Then, the cells and GS and GS-Cl were co-cultured with 0–15 mg mL^−1^ for 12 h at 37 °C and 5% CO_2_. After 12 h incubation, 90 μL of culture medium and 10 μL of CCK-8 solution were added to each well. Then, the plates were incubated for another 2 h in the same conditions. Finally, cell viability was then evaluated by using a spectrophotometric microplate reader (Bio-Rad 680, Hercules, CA, USA) to measure the optical density value at 450 nm. The blank group was a 100 μL culture medium, and the culture solution with GS was added as control groups. Cell viability was calculated by Equation (4) below:

(4) $$Cell\;Viability\left(\%\right)=\frac{\left(OD-{OD}_{blank}\right)}{\left({OD}_{GS}-{OD}_{blank}\right)}\times 100\%$$

where *OD* is the value of the experimental groups, *OD_blank_* is the value of the blank group, and *OD_GS_* is the value of the control group.

### 2.9. In Vivo Wound Healing Study

To evaluate the antibacterial activity of GS-Cl for wound healing in vivo, the *S. aureus* and *E. coli* infected murine model was used. The BALB/c mice (6–8 weeks) were randomly divided into six groups, namely (1) GS-Cl-0.03, (2) GS-Cl-0.05, (3) GS-Cl-0.1, (4) GS-Cl-0.3, (5) Tegaderm film (control), and (6) GS (blank). Circular wounds with a 10 mm diameter were made on the backs of the mice by using a surgical scalpel. Then, the *S. aureus* and *E. coli* suspension (50 μL 2 × 10^5^ CFU mL^−1^) was added to each wound and covered with Tegaderm^3M^ film for 2 days to establish the models. The Tegaderm^3M^ film and different gelatin sponges were covered on the wounds and changed every 2 days. The wound areas were recorded by pictures every 2 days, and the closure ratio was calculated by Equation (5) below:

(5) $$Wound\;area\;closure\left(\%\right)=\left(1-\frac{S_{t}}{S_{0}}\right)\times 100\%$$

where *S*_0_ is the initial wound area and *S_t_* is the real-time wound area. On the 4th day, the bacteria on the wound were collected by sterile cotton swabs. After dilution, the bacteria were dispersed on the LB agar and incubated for counting. On the 10th day and the 16th day, the wound tissues were excised for staining after all the mice were euthanized.

## 3. Results and Discussion

### 3.1. The Influence of Chlorination Time and NaClO Concentrations on the Chlorine Contents

**Figure 1:**
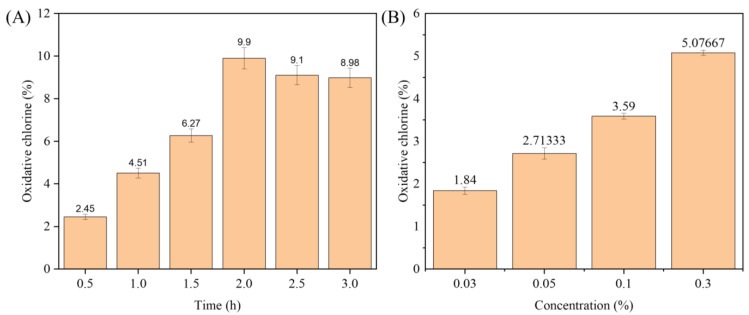
Effect of (**A**) N-chlorination time, and (**B**) NaClO concentration on oxidative chlorine content loaded onto GS.

To optimize the N-chlorination reaction, N-chlorinations were performed using different NaClO concentrations and chlorination times. Figure 1A showed the N-chlorination time effects on the oxidative chlorine load (Cl^+^%), while other conditions like concentrations were the same. The Cl^+^% increases gradually from 2.45 at the original time, and after 2 h, it almost keeps a balance value at about 9. This might be attributed to the fixed amount of active group NH_2_ on the gelatin sponge, leading to the steady value of oxidative chlorine. Figure 1B shows that a sodium hypochlorite solution concentration affects the oxidative chlorine load (Cl^+^%) when the chlorination time was fixed at 2 h, which tends to increase sharply in the concentration range of 0.03~0.3 wt% with about 1.8 to 5.0 Cl^+^%. However, when the concentration exceeds 0.3 wt%, the sponge structure will be destroyed due to the strong oxidation of sodium hypochlorite. These findings suggest the extension of chlorination times, and an increase in NaClO concentrations within a certain range can enhance the loading of oxidative chlorine of the gelatin sponge. Therefore, 2 h of chlorination time with a 0.3 wt% of sodium hypochlorite solution concentration was selected as the optimal condition for future experimental investigation, as represented by GS-Cl-0.3.

### 3.2. FTIR Analysis

**Figure 2:**
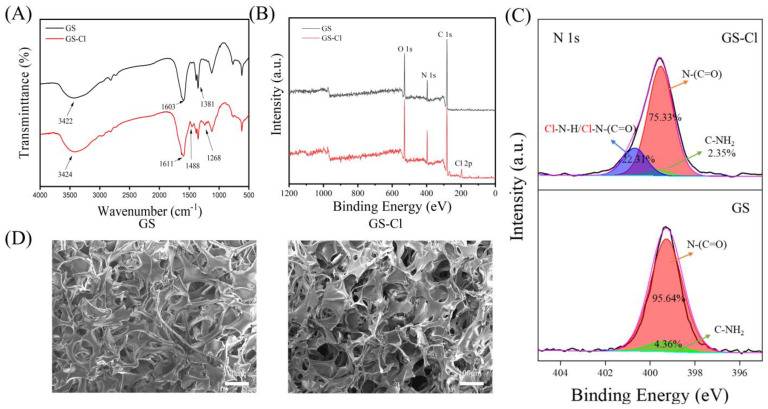
Characterization of GS-Cl. (**A**) FTIR-ATR and (**B**) XPS provide evidence for GS-Cl, (**C**) the precise spectra of N 1s. (**D**) SEM photographs of morphologies of the GS and GS-Cl.

The FTIR spectra of GS and GS-Cl are presented in Figure 2A. It can be obviously seen that there were wide absorption bands that appeared at 3422 cm^−1^ and 3424 cm^−1^ for GS and GS-Cl, respectively, which was attributed to the overlapping of O-H and N-H stretching vibration [32]. The peaks of GS at 1603 and 1381 cm^−1^ were assigned to C=O stretching and the C-N in-plane bending of amide, and the decrease in the absorption peak area at 1381 cm^−1^ for GS-Cl was supported by the conversion of N-H to N-Cl, confirming the successful chlorination [33]. At the same time, there were two additional absorption peaks that appeared at 1488 cm^−1^ and 1268 cm^−1^ in GS-Cl, which were attributed to the N-H stretching in the Cl-N-H and in the Cl-N-(C=O) groups after chlorination, respectively. This consequence further indicated the successful chlorination process. The C=O stretching of the amide of GS was found at 1603 cm^−1^, and the absorption peak of the C=O shifts from 1603 cm^−1^ to 1611 cm^−1^ after chlorination. This result is due to the oxidizing chlorine’s electron-withdrawing effect.

### 3.3. XPS Analysis

XPS analysis was used to further characterize the presence of the chemical elements of GS and GS-Cl-0.3 for confirmation of the chlorination process. The presence of these elements can be attributed to different components, including C, N, and O, all originating from the gelatin skeleton. Cl arises from the reaction between the ClO^−^ and N-H bond of the gelatin sponge. The same characteristic peaks of C 1s, O 1s, and N 1s were clearly present for both GS and GS-Cl-0.3 in Figure 2B. Compared to the spectrum of GS, that of GS-Cl-0.3 supplied an additional absorption band at 198 eV and 269 eV, corresponding to Cl 2p and Cl 2s, which well-verified the existence of chlorine atoms on GS-Cl-0.3. In addition to confirming the conversion of N-H to N-Cl, the precise spectra of N 1s were determined in Figure 2C. It showed that the GS sample has two main types of N, -N-(C=O) and -C-NH_2_, at about 399.3 eV and 399.6 eV, with 96.64% and 4.36%, respectively. While in the GS-Cl sample, there was an additional peak with a percentage of 22.31% at about 400.5 and 400.6 eV, which means the Cl-N-H and Cl-N-(C=O), respectively. However, these two binding energies were too close to divided. In addition, the percentages of -N-(C=O) and -C-NH_2_ declined to 75.33% and 2.35%, respectively. These results indicated that the amine and amide groups were chlorinated to N-Cl.

### 3.4. SEM Analysis

The morphologies of the GS and GS-Cl-0.3 were investigated by SEM, with the results in Figure 2D. Notably, both GS and GS-Cl-0.3 showed the same porous structure, and the GS-Cl-0.3 did not exhibit other changes. It indicated that the structure of GS was not destroyed after N chlorination. Based on the aforementioned test results, the GS-Cl-0.3 structure can be described as a porous material with the addition of Cl through the conversion of N-H to N-Cl.

### 3.5. Mechanical and Stability Studies

**Figure 3:**
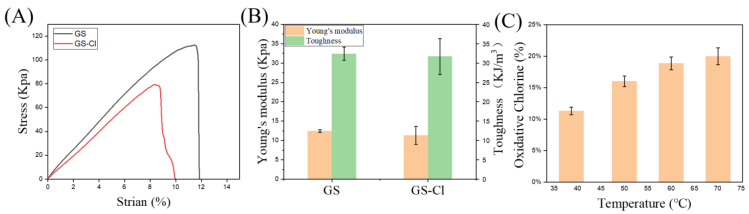
Images of GS and GS-Cl under stretching (**A**) Young’s modulus and (**B**) toughness. (**C**) Effect of temperature on oxidative chlorine released by GS-Cl.

The mechanical properties of GS and GS-Cl were determined in Figure 3. As shown in Figure 3A, the GS and GS-Cl exhibited a moderate Young’s modulus, with 12.42 and 11.27 kPa, respectively. And the fracture was observed when they were stretched by about 110%. In Figure 3B, after chlorination, the GS and GS-Cl both exhibited similar Young’s moduli and toughness, with approximately 12 kPa and 32 kJ/m^3^, respectively. These results demonstrated that the mechanical properties were not changed after chlorination, which implies the high stability of this concentration. In addition, Figure 3C shows the effect of different temperatures on releasing oxidative chlorine in the same period. At 38.6 °C, it released about 11.32 Cl^+^%, and by heating, the Cl^+^% was increased to about 16.03, 18.86, and 20.00, respectively, which indicates that Cl^+^ was released faster when the temperature was increased. However, the structure of GS-Cl was destroyed above 70 °C in about 5 min.

### 3.6. Antibacterial Activity

**Figure 4:**
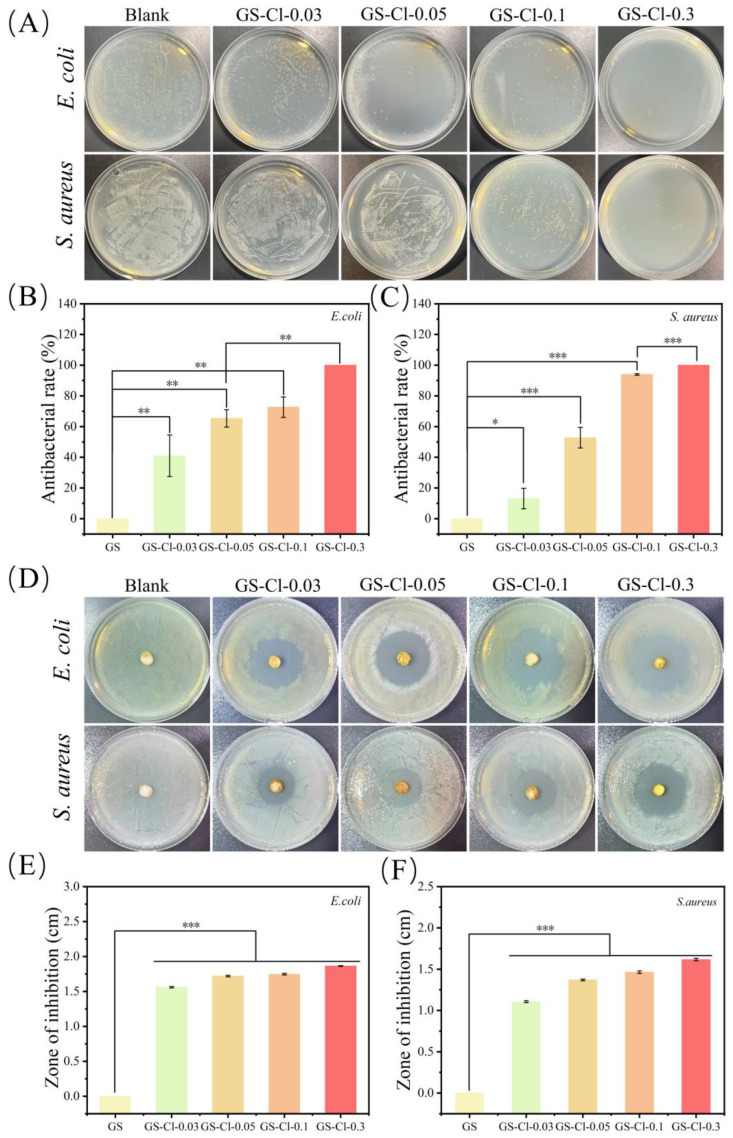
Antimicrobial properties of GS-Cl. (**A**) Evaluation of *E. coli* and *S. aureus* growth by the spread plate method. (**B**,**C**) Antibacterial rate of GS-Cl against *S. aureus* and *E. coli*. (**D**) Optical images of the inhibition zone for GS. (**E**,**F**) Quantitative results of the inhibition zones. and GS-Cl against *S. aureus* and *E. coli*. Independent sample T test was used for statistical analysis of quantitative data. For the test, *p* < 0.05 was considered statistically significant. *, *p* < 0.1; **, *p* < 0.01; ***, *p* < 0.001.

The in vitro antimicrobial activities of GS and GS-Cl were determined with *S. aureus* and *E. coli*. In Figure 4A, on the blank plate, the selected two bacteria, *S. aureus* and *E. coli,* appeared as dense survival, indicating that GS had no antibacterial activities. The number of bacterial colonies in GS-Cl-0.03, GS-Cl-0.05, GS-Cl-0.1, and GS-Cl-0.3 decreased significantly compared with the blank. The antibacterial efficiency was quantitatively analyzed in Figure 4B,C, which revealed that the inhibition rates of GS, GS-Cl-0.03, GS-Cl-0.05, GS-Cl-0.1, and GS-Cl-0.3 for *E. coli* were 0%, 41%, 65.33%, 72.67%, and 100%, respectively. The inhibition rates of GS, GS-Cl-0.03, GS-Cl-0.05, GS-Cl-0.1, and GS-Cl-0.3 for *S. aureus* were 0%, 13.11%, 52.78%, 93.96%, and 100%. The test results of the two different bacteria indicated the antibacterial activity of GS-Cl would enhance with NaClO concentration increases. Furthermore, the antimicrobial activity of the sample was evaluated through the zone of inhibition test, with the results in Figure 4D. There was no inhibition zone against bacteria in the GS, and GS-Cl-0.03, GS-Cl-0.05, GS-Cl-0.1, and GS-Cl-0.3 individually formed an obvious growth-inhibition zone. This result implies that the released active chlorines from GS-Cl have efficiently killed the bacteria. As shown in Figure 4E,F, the diameters of the inhibition zone for GS, GS-Cl-0.03, GS-Cl-0.05, GS-Cl-0.1, and GS-Cl-0.3 against *E. coli* were 0, 1.56, 1.72, 1.75, and 1.86 cm, respectively. The diameters of the inhibition zone for GS, GS-Cl-0.03, GS-Cl-0.05, GS-Cl-0.1, and GS-Cl-0.3 against *S. aureus* were 0, 1.11, 1.37, 1.46, and 1.62 cm, respectively. The results indicated that GS-Cl-0.3 possessed the best bacterial inhibition capacity compared to GS-Cl-0.03, GS-Cl-0.05, and GS-Cl-0.1. It was due to that, among these four kinds of samples, GS-Cl-0.3 could release the highest concentration of active chlorines at the same time, which could effectively kill the bacteria.

**Figure 5:**
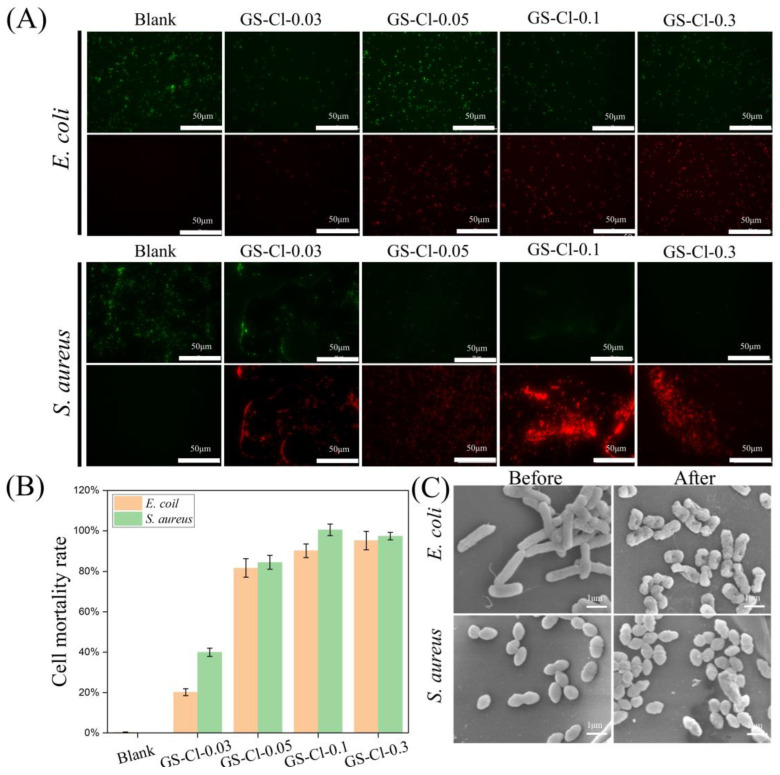
(**A**) Fluorescent staining images of *E. coli* and *S. aureus* after treatment with GS-Cl. (**B**) Mortality rate of *E. coil* and *S. aureus* calculated by ImageJ (**C**) SEM images of *E. coli* and *S. aureus* after treatment with GS and GS-Cl.

The live/dead bacterial staining test was determined to support the above results. SYTO9 can enter all bacteria and present as green fluorescence, while PI can only enter the dead bacteria with damaged cell membranes and present as red fluorescence. As shown in Figure 5A, the *E. coli* and *S. aureus* in GS-Cl-0.3 were almost fluoresced red, which corresponds to 100 percent dead cells, but there were almost no bacteria fluoresced red in the GS. In addition, the mortality rates of both *E. coli* and *S. aureus* were calculated by ImageJ 1.52 p. As shown in Figure 5B, for *E. coli*, the cell mortality rate in the blank group was 0.27%, while the highest rate reached 95.19% in the GS-Cl-0.3 group, and those of the other groups were 20.21%, 81.66%, and 90.13%, respectively. For *S. aureus*, there was no mortality in the blank group, and the bacteria were almost dead in the GS-Cl-0.1 group. Meanwhile, those of other groups were 39.98%, 84.49%, and 97.39%, respectively. These results were the same as the live/dead bacterial staining assay, which further indicated that the higher oxidative chlorine the samples have, the better their antibacterial properties are.

In addition, the morphology of the bacteria treated by the GS-Cl was recorded by SEM to further confirm their antibacterial properties. GS-Cl-0.3 was selected as a representative chlorinated sponge due to its best antibacterial activity. The result in Figure 5C demonstrated that, after GS treatment, both *E. coli* and *S. aureus* were intact and showed smooth and intact cell walls, *E. coli* had a rodlike shape, and *S. aureus* had a round shape. After being in contact with GS-Cl-0.3 for 6 h, *E. coli* and *S. aureus* both displayed cell deformation and surface collapse. These results suggested that the GS-Cl could destroy the structures of bacteria, owing to chloramine-induced damage.

### 3.7. Biocompatibility

**Figure 6:**
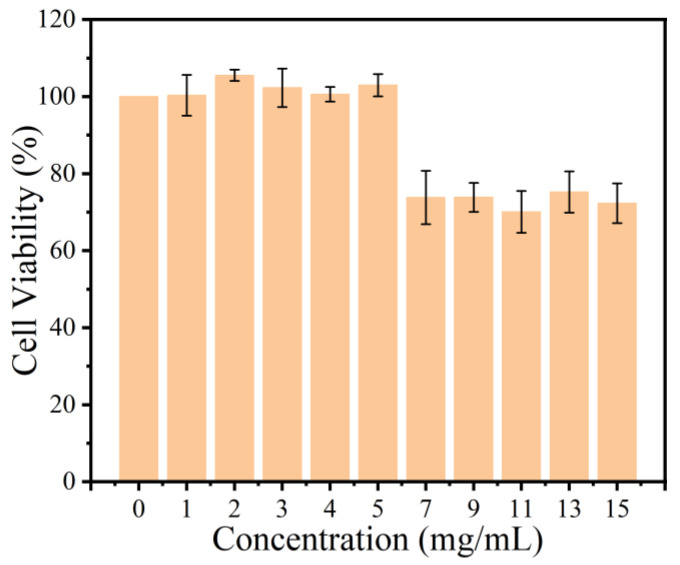
Cell viability of Hacat cells cultured on different concentrations of GS-Cl for 12 h.

Biocompatibility is also essential before applying the GS-Cl for an in vivo test. Thus, we evaluated the cell viability of Hacat cells by a CCK-8 experiment after incubation with GS and GS-Cl-0.3 in Figure 6. The result indicated that the cell viabilities of any of the GS-Cl samples were all above 75% with the concentration range of 0–15 mg/mL, and there was no cytotoxic behavior found during the culture when the concentration was lower than 5 mg/mL. No cytotoxic behavior is due to the fact that the gelatin is a kind of protein that has intrinsic biocompatibility, and the toxicity of Cl^+^ causes the decline of cell viability above 5 mg/mL. According to ISO10993-5, cell viability over 70% is considered to show samples are non-toxic, meaning that GS-Cls are biocompatible, which supplies support for potential biomedical applications.

### 3.8. In Vivo Infectious Wound Healing Studies

**Figure 7:**
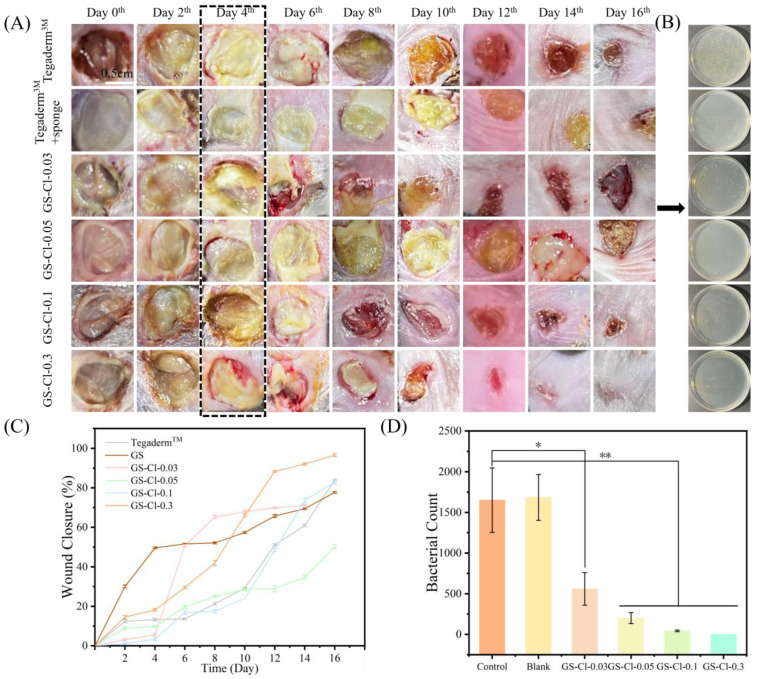
In vivo infectious mouse wound healing and antimicrobial studies. The *S. aureus* infected wound was established after contacting *S. aureus* for two days. (**A**) Photographs of infected wounds at a given time. (**B**) Optical images of the colony of bacteria from the wound on the plates. (**C**) Wound area closure ratio during the wound healing. (**D**) Number of bacterial colonies in the wound. Independent sample T test was used for statistical analysis of quantitative data. For the test, *p* < 0.05 was considered statistically significant. *, *p* < 0.1; **, *p* < 0.01.

Based on the above experiments, the in vitro results showed that the GS-Cl with good antibacterial activity and biocompatibility demonstrated potential application in wound infections. Therefore, the skin wound (1 cm) mice model that was infected by *S. aureus* and *E. coli* was established to further evaluate the in vivo feasibility of GS-Cl. All mice were randomly divided into six groups after being infected, namely (1) GS-Cl-0.03, (2) GS-Cl-0.05, (3) GS-Cl-0.1, (4) GS-Cl-0.3, (5) Tegaderm film (control), and (6) GS (blank). From Figure 7A, the wounds of all the mice showed inflammation and purulency after *S. aureus* infection (0th day). In the control and blank groups, the wound area became larger, and the infection was worse on the 6th day due to the growth of bacteria. However, the treated groups could obviously reduce pus and inflammation. It indicated that the GS has no antibacterial activity, and it can provide more suitable conditions for the growth of bacteria by water-absorbing and moisturizing, resulting in slow wound healing. On the 16th day, wounds treated with GS-Cl-0.3 were almost completely healed, which is the fastest compared to the control group and that treated by other groups. Figure 7C shows the change in wound closure over a 16-day period. After 16 days of healing, the wound treated with GS-Cl-0.3 achieved significant closure to 96.59%, while the others treated with GS-Cl-0.1, GS-Cl-0.05, GS-Cl-0.03, GS, and the bared wound showed around 82.85%, 50.23%, 71.18%, 77.72%, and 83.93% wound closure, respectively. To test whether bacteria were left in the wound, bacteria were collected on the 4th day(shown in Figure 7A, the dashed line box). As shown in Figure 7B,D, compared with the GS and bared wound, the wounds treated with GS-Cl-0.03, GS-Cl-0.05, GS-Cl-0.1, and GS-Cl-0.3 had much fewer bacteria, and the counts of surviving bacterial colonies were decreased with the rising of the loading of oxidative chlorine. The wound treated with GS-Cl-0.3 had no bacteria, which was due to the good antibacterial property of GS-Cl by releasing the largest concentration of oxidative chlorine. These results were consistent with the in vitro antimicrobial activity test, which suggested that the GS-Cl samples have great antimicrobial properties.

**Figure 8:**
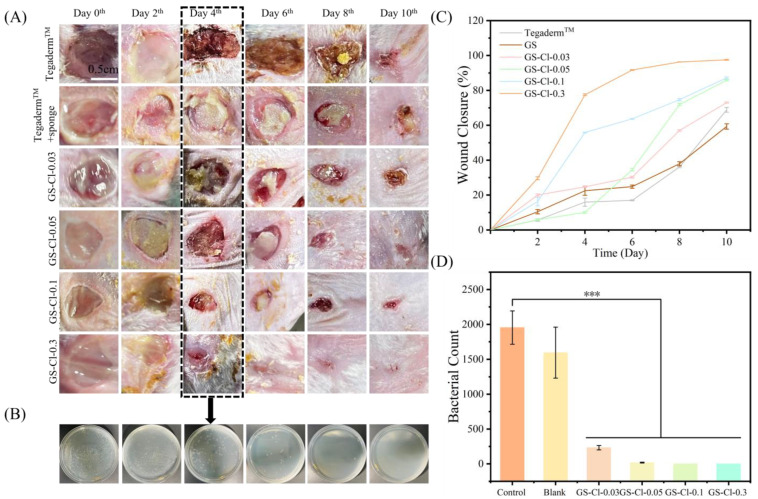
In vivo infectious mouse wound healing and antimicrobial studies. The *E. coli*-infected wound was established after contacting *E. coli* for two days. (**A**) Photographs of infected wounds at a given time. (**B**) Optical images of the colony of bacteria from the wound on the plates. (**C**) Wound area closure ratio during the wound healing. (**D**) Number of bacterial colonies in the wound. Independent sample T test was used for statistical analysis of quantitative data. For the test, *p* < 0.05 was considered statistically significant. ***, *p* < 0.001.

The treatment of the *E. coli*-infected skin wound (1 cm) mice model is in Figure 8A. On the 6th day, it is obvious that the wounds treated with the GS-Cl (especially for the GS-Cl-0.3) shrink, while wounds of the control and blank groups heal slowly. It was clearly seen that the GS-Cl-0.3 treated wounds were almost closed on the 10th day, while the other groups remained unhealed. The change in wound closure over a 10-day period is shown in Figure 8C. The wound closure rate of the GS-Cl-0.3 group was 97.50%, which was dramatically higher than those of the other groups, with 87.16%, 85.79%, 72.99%, 59.31%, and 68.85%, respectively. In addition, we evaluated the surviving wound bacterial colonies from different treatments on the 4th day(shown in Figure 8A, the dashed line box). From Figure 8B, there were few bacterial colonies in the groups GS-Cl-0.1 and GS-Cl-0.3, but a large number of bacteria colonies were in the control and blank groups. According to colony count statistics, the number of bacteria in GS-Cl-0.3 was significantly lower than that of the other groups in Figure 8D, which agreed well with the in vitro antibacterial performance. The quantitative analysis further confirmed that GS-Cl-0.3 has better antibacterial effects.

**Figure 9:**
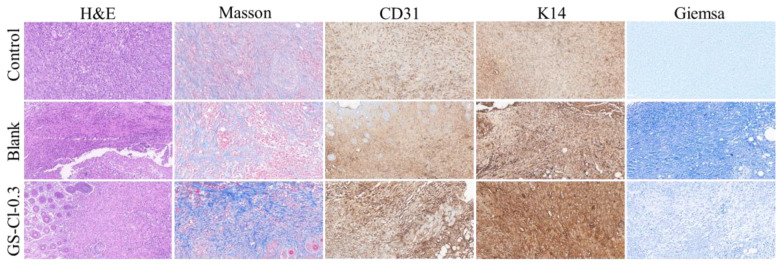
Histological analysis of skin wound infected by *E. coli* in mice: H&E staining, Masson staining, CD31 immunohistochemistry, K14 staining, and Giemsa staining.

**Figure 10:**
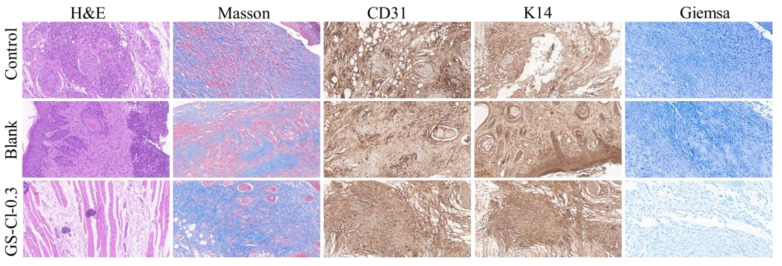
Histological analysis of *S. aureus* skin infection wound in mice: H&E staining, Masson staining, CD31 immunohistochemistry, K14 staining, and Giemsa staining.

There are three important stages during the healing process, namely inflammation, hyperplasia, and remodeling. Histological analysis was performed by H&E, Masson, Giemsa, CD31, and K14 to evaluate the skin regeneration at the wound site. As shown in Figure 9 and Figure 10, there were fewer inflammatory cells in the GS-Cl-0.3 group than the control group in H&E, indicating that the sample had a positive influence on skin regeneration. Collagen plays an important role in the processing of wound healing as a skeleton for new tissue, which is crucial to high strength. Therefore, the content of collagen can also be used to evaluate the wound healing effect. The Masson results showed that there were more significant enhancements in the collagen staining intensity on the wound of the GS-Cl-0.3 group. However, the collagen structure of the control group and the blank one were relatively loose and disordered. The immunohistochemical staining of CD31 showed that the expression of CD31 was enhanced in the GS-Cl-0.3 group, indicating that the stimulation of angiogenesis in the GS-Cl-0.3 group was stronger in the process of wound healing.

## 4. Conclusions

In conclusion, we have reported a straightforward approach for the preparation of a set of N-Cl bond-containing N-halamine proteins with GS as a model and Cl^+^ as an antibacterial component by chlorinated amine treatment. Its antibacterial activities are mainly based on the release of active chlorine in water and directly reacts with the vital bacterial cell, destroys cell membranes, enters the microbial body, and interferes with cellular enzyme activity and metabolic processes, resulting in bacterial death. In vitro studies demonstrate that a chlorinated gelatin sponge had an excellent antibacterial effect, both on *E. coli* and *S. aureus*. In vivo, studies indicate that the GS-Cl sample inhibited *E. coli* and *S. aureus* growth and promoted wound healing. These studies show that proteins can be chlorinated and endowed with antimicrobial properties, providing an alternative to antibacterial materials for a wide range of biomedical applications.

## Data Availability

The original contributions presented in the study are included in the article, further inquiries can be directed to the corresponding author.
